# Value of the CHA_2_DS_2_-VASc score and Fabry-specific score for predicting new-onset or recurrent stroke/TIA in Fabry disease patients without atrial fibrillation

**DOI:** 10.1007/s00392-018-1285-4

**Published:** 2018-05-24

**Authors:** Dan Liu, Kai Hu, Marie Schmidt, Jonas Müntze, Octavian Maniuc, Daniel Gensler, Daniel Oder, Tim Salinger, Frank Weidemann, Georg Ertl, Stefan Frantz, Christoph Wanner, Peter Nordbeck

**Affiliations:** 1 Department of Internal Medicine I, University Hospital Würzburg, Oberdürrbacher Str. 6, 97080 Würzburg, Germany; 2 Comprehensive Heart Failure Center (CHFC), University of Würzburg, Würzburg, Germany; 3 Medizinische Klinik I, Klinikum Vest, Recklinghausen, Germany

**Keywords:** Fabry disease, Global systolic strain, Stroke, Transient ischemic attack

## Abstract

**Objectives:**

To evaluate potential risk factors for stroke or transient ischemic attacks (TIA) and to test the feasibility and efficacy of a Fabry-specific stroke risk score in Fabry disease (FD) patients without atrial fibrillation (AF).

**Background:**

FD patients often experience cerebrovascular events (stroke/TIA) at young age.

**Methods:**

159 genetically confirmed FD patients without AF (aged 40 ± 14 years, 42.1% male) were included, and risk factors for stroke/TIA events were determined. All patients were followed up over a median period of 60 (quartiles 35–90) months. The pre-defined primary outcomes included new-onset or recurrent stroke/TIA and all-cause death.

**Results:**

Prior stroke/TIA (HR 19.97, *P* < .001), angiokeratoma (HR 4.06, *P* = .010), elevated creatinine (HR 3.74, *P* = .011), significant left ventricular hypertrophy (HR 4.07, *P* = .017), and reduced global systolic strain (GLS, HR 5.19, *P* = .002) remained as independent risk predictors of new-onset or recurrent stroke/TIA in FD patients without AF. A Fabry-specific score was established based on above defined risk factors, proving somehow superior to the CHA_2_DS_2_-VASc score in predicting new-onset or recurrent stroke/TIA in this cohort (AUC 0.87 vs. 0.75, *P* = .199).

**Conclusions:**

Prior stroke/TIA, angiokeratoma, renal dysfunction, left ventricular hypertrophy, and global systolic dysfunction are independent risk factors for new-onset or recurrent stroke/TIA in FD patients without AF. It is feasible to predict new or recurrent cerebral events with the Fabry-specific score based on the above defined risk factors. Future studies are warranted to test if FD patients with high risk for new-onset or recurrent stroke/TIA, as defined by the Fabry-specific score (≥ 2 points), might benefit from antithrombotic therapy. *Clinical trial registration* HEAL-FABRY (evaluation of HEArt invoLvement in patients with FABRY disease, NCT03362164).

**Electronic supplementary material:**

The online version of this article (10.1007/s00392-018-1285-4) contains supplementary material, which is available to authorized users.

## Introduction

Fabry disease (FD) is an X-linked inherited disorder characterized by deficiency of lysosomal α-galactosidase A, which affects many body organs including the skin, eyes, gastrointestinal system, kidney, heart, peripheral and central nervous system due to progressive accumulation of globotriaosylceramide (Gb3) and other glycosphingolipids within lysosome carrying cells [1–3]. Patients with FD often experience cerebrovascular events such as stroke or transient ischemic attacks (TIA) at young age, even before FD diagnosis and in the absence of other clinical events. Reported incidence of stroke ranged from 7 to 48% in FD patients [4, 5]. The “silent progression” nature of stroke in a relevant portion of FD patients thus requires timely recognition, risk stratification and efficient treatment. A better understanding of the risk factors for stroke in patients with FD might lead to improved prevention and therapeutic strategies not only in FD patients, but potentially also in other multisystemic diseases, storage disorders, and/or (secondary) cardiomyopathies. Currently, the risk factors of stroke/TIA in FD patients without AF remain largely unknown.

Numerous scoring methods have been established for prediction of stroke in patients with atrial fibrillation (AF) [6], including the CHADS_2,_ CHA_2_DS_2_-VASc, or the ATRIA (Anticoagulation and Risk Factors in Atrial Fibrillation) risk score [7]. The CHA_2_DS_2_-VASc score is currently the most commonly used score for estimating the risk of stroke in patients with non-rheumatic AF [8]. Clinical utility of this risk score was also suggested in heart failure patients with reduced ejection fraction without AF [9, 10]. It remains unknown now if the CHA_2_DS_2_-VASc score can also be meaningfully used to predict stroke risk in patients with inherited diseases with myocardial damage and cardio-cerebral vascular complications, such as FD.

Congestive heart failure patients face an increased risk of stroke [11]. It is thus reasonable to speculate that conventional and modern left ventricular (LV) functional markers derived from echocardiography, i.e., ejection fraction (EF) and global systolic strain (GLS) might be useful to predict stroke risk in FD patients. In the present study, we tested the hypothesis that a Fabry-specific score based on pre-defined risk factors for stroke/TIA might be feasible to evaluate the risk of stroke in FD patients without AF. The purposes of this study were (i) to determine potential risk factors for stroke/TIA in FD patients without AF; (ii) to evaluate whether the CHA_2_DS_2_-VASc score can predict cerebrovascular events in FD patients without AF; (iii) to develop and test the feasibility and efficacy of a modified Fabry-specific risk score method based on defined risk factors in a large FD patient cohort.

## Methods

### Study population

This retrospective investigation on stroke/TIA risk of FD patients included patients from a prospective single-center observational study (HEAL-FABRY, ClinicalTrials.gov Identifier: NCT03362164). In brief, patients with genetically confirmed FD referred to the Fabry Center for Interdisciplinary Treatment Würzburg (FAZIT) between 2001 and June 2015 were eligible for this study; all patients with follow-up for > 2 years were screened. Sixteen patients with AF were excluded and 159 patients were included for the final analysis (Suppl. Fig. 1). All patients underwent extensive on-site characterization of disease severity, related organ involvement and concomitant diseases. Demographic data, medical history and laboratory data were collected from the annual visit at FAZIT including review of all related external medical records. Conventional echocardiographic measurements and speckle tracking imaging (STI) analysis were performed off-line by two experienced investigators. The study was conducted in accordance to the Declaration of Helsinki, and approved by the local Ethics Committee at the University of Würzburg. Written informed consent was obtained from all patients.

### Stoke and TIA diagnosis

Prior history of stroke and TIA were identified from medical records at baseline visit. New-onset or recurrent stroke was defined according to the AHA/ASA definition of stroke in 2013, based on pathological, imaging or other objective evidence of infarction. In case of the absence of this evidence, the persistence of symptoms of at least 24 h or until death remained a method to define stroke [12]. TIA was defined as a transient episode of neurological dysfunction caused by focal brain, spinal cord, or retinal ischemia, with typical clinical symptoms and without evidence of acute infarction, based on a comprehensive evaluation including clinical, laboratory, and imaging modalities [13].

### CHA_2_DS_2_-VASc score

The CHA_2_DS_2_-VASc score was calculated based on the original description by Lip et al. [8].

### Electrocardiogram

Standard resting 12-channel ECG was performed in all patients. A total of 134 (84.3%) patients received a 24-h Holter ECG monitoring at baseline visit. An atrial high-rate episode was defined as an atrial tachyarrhythmia with an atrial rate ≥ 180 beats/min lasting ≥ 5 min.

### Conventional and speckle tracking echocardiography

A standard echocardiographic examination was performed on a Vivid 7/9e echo machine (GE Vingmed, Horten, Norway). Cardiac structural and functional measurements were performed off-line after initial image acquisition according to recommended guidelines [14]. STI analysis was performed using EchoPAC software (GE, Horten, Norway) [15]. Global longitudinal peak systolic strain (GLS) was calculated by averaging strain values of all LV 18 segments.

### Cardiac magnetic resonance imaging

Cardiac magnetic resonance (CMR) imaging was performed using a 1.5 T full body magnetic resonance scanner (Magnetom Symphony Quantum/Avanto, Siemens Medical Systems, Erlangen, Germany) as previously described [16]. Late gadolinium enhancement (LGE) images were acquired 15 min after intravenous injection of 0.2 mmol/kg gadopentetate dimeglumine, using T1-weighted inversion recovery imaging sequences (field of view 240 × 320 mm^2^, matrix size 165 × 256, slice thickness 8 mm, echo time 3.4 ms, repetition time 7.5 ms). A stack of multiple short-axis views covering the entire left ventricle were applied to detect changes in the LV myocardial tissue. All LV segments were evaluated for the presence of myocardial fibrosis by two radiologists who were blinded to all clinical data. Areas with signal intensity above the average of the normal myocardium plus 2 standard deviations were defined as LGE positive. For analysis of right ventricular (RV) function, short-axis views covering the RV were analyzed with a commercially available post-processing software for CMR (CVI 42®, Circle Cardiovascular Imaging Inc., Calgary, Canada). For the quantitative assessment of the RVEF, the RV volume was determined in systole and diastole.

### Diagnosis of Fabry cardiomyopathy

Diagnostic criteria by echocardiography or/and CMR on Fabry cardiomyopathy were as follows: (1) LV concentric hypertrophy with a LV wall thickness ≥ 12 mm or asymmetric left ventricular hypertrophy (LVH) characterized by significant thickening at the septum and thinning at the posterior wall, detected by echocardiography and CMR; (2) reduced LVEF (< 55%); (3) myocardial replacement fibrosis detected by CMR LGE imaging. Genetically proven FD patients who matched any of these three criteria were defined as Fabry cardiomyopathy [17].

### Statistical analysis

Continuous variables are expressed as mean ± standard deviation or median (quartiles). Categorical variables are presented as count and percentage. Differences on continuous data between groups were compared using unpaired Student’s *t* test or Mann–Whitney *U* test, as appropriate. Categorical data were compared using a similar approach employing Chi-square or Fisher’s exact test. Given cumulative hazard analyses, Kaplan–Meier curves were plotted and compared using the log rank test. All variables with *P* < .05 in Tables 1 and 2 were tested as potential risk factors of new-onset or recurrent stroke/TIA in the univariable Cox proportional hazard regression models as well as in the multivariable models adjusted for age and sex, presented as the hazard ratio (HR) with 95% confidence intervals (CI). The cut-off values of creatinine, left ventricular posterior wall thickness (LVPWd) and GLS for predicting new-onset or recurrent stroke/TIA events were derived from receiver operating characteristic curves analysis by maximizing the sum of the sensitivity and specificity. The significance of the difference between the areas under two receiver operating characteristic curves was compared using Hanley and McNeil’s method. Statistical significance was defined as *P* < .05 (two-tailed test). Statistical analysis was performed using IBM SPSS, version 23 for Windows (IBM Corp., New York, USA).

**Table 1:** Clinical, ECG, CMR characteristics in FD patients with and without stroke/TIA events

	Total	No events	Prior/recurrent/new-onset events	*P* value
	*N* = 159	*N* = 121	*N* = 38	
Age at baseline examination (years)	40 ± 14	39 ± 15	45 ± 13	0.020
Age at first diagnosis (years)	38 ± 15	37 ± 15	42 ± 13	0.011
Age occurred events (years)	40 ± 13 Range 14–68	–	40 ± 13 Range 14–68	–
Male/female [*n* (%)]	70/89 (42.1/57.9)	51/70 (42.6/57.4)	19/19 (50.0/50.0)	0.395
BMI (kg/m^2^)	23 ± 4	23 ± 5	23 ± 4	0.904
Heart rate (beats/min)	67 ± 13	66 ± 13	67 ± 14	0.694
Systolic blood pressure (mmHg)	125 ± 20	124 ± 20	125 ± 19	0.804
Diastolic blood pressure (mmHg)	80 ± 13	80 ± 13	81 ± 12	0.478
NYHA class III–IV [*n* (%)]	18 (11.3)	9 (7.4)	9 (23.7)	0.015
Medical history [*n* (%)]				
Diabetes	2 (1.3)	1 (0.8)	1 (2.6)	0.422
Hypertension	51 (32.1)	36 (29.8)	15 (39.5)	0.263
Smoking	36 (22.6)	28 (23.1)	8 (21.1)	0.788
Arrhythmia	15 (9.4)	11 (9.1)	4 (10.5)	0.757
Coronary heart disease	11 (6.9)	5 (4.1)	6 (15.8)	0.023
Myocardial infarction	3 (1.9)	1 (0.8)	2 (5.3)	0.142
Chronic kidney disease > stage II	32 (20.1)	22 (18.2)	10 (26.3)	0.275
Dialysis	11 (6.9)	8 (6.6)	3 (7.9)	0.725
Kidney transplantation	5 (3.1)	3 (2.5)	2 (5.3)	0.594
Fabry associated [*n* (%)]				
Angiokeratoma	59 (37.1)	39 (32.2)	20 (52.6)	0.023
Hearing loss	27 (17.0)	18 (14.9)	9 (23.7)	0.207
Dysarthria	8 (5.1)	4 (3.3)	4 (10,8)	0.088
Vertigo	24 (26.7)	19 (27.5)	5 (23.8)	0.735
Tinnitus	52 (32.9)	38 (31.7)	14 (36.8)	0.554
Depression	17 (10.8)	11 (9.1)	6 (16.2)	0.233
Frequent diarrhea	50 (31.4)	41 (33.9)	9 (23.7)	0.237
Chronic pain	45 (28.7)	32 (26.7)	13 (35.1)	0.319
Frequent use of analgesics	27 (17.1)	18 (14.9)	9 (24.3)	0.181
Blood tests				
Lyso-Gb3 (ng/ml)	9.5 (4.6–25.2)	9.2 (4.5–24.5)	12.3 (4.7–30.8)	0.505
Creatinine (mg/dl)	0.8 (0.7-1.0)	0.8 (0.7-1.0)	0.9 (0.8–1.1)	0.011
GFR (DTPA) (ml/min/1.73 m^2^)	102 (84–123)	104 (88–124)	91 (78–120)	0.111
Hemoglobin (g/dl)	13.6 (12.6–14.7)	13.7 (12.8–14.7)	13.2 (12.1–14.1)	0.111
NT-proBNP (pg/ml)	123 (44–348)	110 (42–309)	185 (66-1228)	0.067
Enzyme replacement therapy [*n* (%)]	91 (57.2)	66 (54.5)	25 (65.8)	0.222
Medication [*n* (%)]				
Angiotensin-converting-enzyme inhibitor/AT1-antagonist	43 (27.0)	31 (25.6)	12 (31.6)	0.471
β-blockers	31 (19.5)	21 (17.4)	10 (26.3)	0.224
Diuretic	16 (10.1)	8 (6.6)	8 (21.1)	0.010
Antithrombotic drugs	9 (5.7)	2 (1.7)	7 (18.4)	0.001
Fabry cardiomyopathy [*n* (%)]	80 (50.3)	55 (45.5)	25 (65.8)	0.029
Clinical outcome				
Follow-up period (from baseline echo to last follow-up visit, months)	63 ± 33 Median 60 (35–90)	62 ± 31 Median 59 (35–89)	64 ± 36 Median 63 (40–93)	0.822
All-cause death [*n* (%)]	11 (6.9)	4 (3.3)	7 (18.4)	0.004
Cardiac death [*n* (%)]	7 (4.4)	2 (1.7)	5 (13.2)	0.009
ECG				
Sinus rhythm [*n* (%)]	152 (95.6)	118 (97.5)	34 (89.5)	0.057
Pacemaker [*n* (%)]	7 (4.4)	3 (2.5)	4 (10.5)	
P duration (ms)	97 ± 19	98 ± 18	95 ± 21	0.373
PQ interval (ms)	141 ± 29	139 ± 26	149 ± 38	0.084
QRS width (ms)	97 ± 23	96 ± 23	99 ± 25	0.429
QT/ QTc duration (ms)	396 ± 40/414 ± 39	395 ± 35/410 ± 35	397 ± 55/424 ± 50	0.796/0.061
Sokolow–Lyon index (mm)	29 ± 14	29 ± 13	31 ± 16	0.349
LVH based on Sokolow [*n* (%)]	42 (27.3)	29 (24.6)	13 (36.1)	0.174
ST–T alterations [*n* (%)]	65 (41.9)	44 (37.3)	21 (56.8)	0.036
24 h Holter ECG	*N* = 134	*N* = 101	*N* = 33	
Supraventricular tachycardia [*n* (%)]	12 (9.0)	9 (8.9)	3 (9.1)	1.000
Atrial high-rate episodes [*n* (%)]	2 (1.5)	2 (2.0)	0	1.000
Silent AF episodes	0	0	0	–
CMR	*N* = 136	*N* = 106	*N* = 30	
LVEF (%)	63 ± 8	64 ± 8	62 ± 8	0.330
LVMi (g/m^2^)	82 ± 31	79 ± 26	96 ± 43	0.048
EDVi (ml/m^2^)	76 ± 18	76 ± 17	76 ± 21	0.889
ESVi (ml/m^2^)	28 ± 10	28 ± 10	29 ± 9	0.609
SVi (ml/m^2^)	48 ± 12	48 ± 10	45 ± 17	0.283
CI (l/min/m^2^)	3.1 ± 1.0	3.2 ± 0.7	2.9 ± 1.5	0.185
RVEF (%)	55 ± 10	54 ± 10	56 ± 11	0.312
LGE [*n* (%)]	56 (41.2)	41 (38.7)	15 (50.0)	0.266

*BMI* body mass index, *NYHA* New York Heart Association, *Lyso-Gb3* globotriaosylsphingosine, *GFR (DTPA)* glomerular filtration rate by the 99mTc-DTPA, *NT-proBNP* N-terminal pro b-type natriuretic peptide, *LVH* left ventricular hypertrophy, *AF* atrial fibrillation, *AHRE* atrial high-rate episodes, *CMR* cardiac magnetic resonance, *EF* ejection fraction, *LVMi* normalized left ventricular mass, *EDVi* normalized left ventricular end-diastolic volume, *ESVi* normalized left ventricular end-systolic volume, *SVi* normalized stroke volume, *CI* cardiac index, *LGE* late gadolinium enhancement

**Table 2:** Echocardiographic data in patients with and without stroke/TIA events

	Total	No events	Prior/recurrent/new-onset events	*P* value
	*N* = 159	*N* = 121	*N* = 38	
LVEDD (mm)	47 ± 6	47 ± 5	47 ± 6	0.890
LVFS (%)	37 ± 6	38 ± 6	38 ± 6	0.889
IVSd (mm)	10.8 ± 3.0	10.1 ± 2.6	12.2 ± 3.4	0.001
LVPWd (mm)	10.4 ± 2.7	9.8 ± 2.4	11.6 ± 3.0	0.002
LVMi (g/m^2^)	103 ± 40	94 ± 34	122 ± 50	0.002
LVEF (%)	65 ± 8	66 ± 7	65 ± 8	0.764
LVEDV (ml)	85 ± 29	86 ± 29	85 ± 32	0.847
LVESV (ml)	31 ± 15	30 ± 15	30 ± 16	0.873
Septal MAPSE (mm)	11.7 ± 5.0	12.0 ± 3.1	11.7 ± 8.8	0.827
Lateral MAPSE (mm)	13.5 ± 2.9	14.0 ± 2.4	13.0 ± 3.5	0.114
TAPSE (mm)	22 ± 6	23.0 ± 4.1	21.1 ± 8.9	0.215
RVD_mid (mm)	27 ± 5	27 ± 4	26 ± 5	0.895
RAA (cm^2^)	14 ± 4	14 ± 3	14 ± 4	0.345
LAVi (ml/m^2^)	26 ± 12	24 ± 9	28 ± 14	0.054
E (cm/s)	87 ± 21	86 ± 19	83 ± 20	0.461
E/A ratio	1.4 ± 0.5	1.4 ± 0.5	1.4 ± 0.6	0.610
DT (ms)	213 ± 53	210 ± 48	230 ± 68	0.110
e′ (cm/s)	8.1 ± 3.5	8.5 ± 3.4	7.4 ± 3.9	0.090
E/e′	13.0 ± 7.0	11.7 ± 5.8	14.6 ± 8.9	0.068
SPAP (mmHg)	27 ± 8	26 ± 7	26 ± 10	0.893
Diastolic dysfunction (%)				0.009
Normal	92 (57.9)	79 (65.3)	13 (34.2)	
Mild	35 (22.0)	22 (18.2)	13 (34.2)	
Moderate	27 (17.0)	17 (14.0)	10 (26.3)	
Severe	5 (3.1)	3 (2.5)	2 (5.3)	
GLS (%)	− 16.8 ± 4.3	− 17.7 ± 3.5	− 15.4 ± 4.8	0.009

*LVEDD* left ventricular end-diastolic dimension, *LVFS* left ventricular fractional shortening, *IVSd* end-diastolic interventricular septal thickness, *LVPWd* end-diastolic left ventricular posterior wall thickness, *LVMI* left ventricular mass indexed to body surface area, *LVEF* left ventricular ejection fraction, *LVEDV* left ventricular end-diastolic volume, *LVESV* left ventricular end-systolic volume, *MAPSE* mitral annular plane systolic excursion, *TAPSE* tricuspid annular plane systolic excursion, *RVD_mid* end-diastolic mid-right ventricular diameter, *RAA* end-systolic right atrial area, *LAVi* left atrial volume indexed to body surface area, *E wave* mitral inflow early diastolic filling velocity, *E*/*A ratio* the ratio of mitral inflow early filling velocity to late diastolic filling velocity, *DT* deceleration time of E wave, *e* tissue Doppler derived mitral annular early diastolic velocity, *E*/*e′* the ratio of early diastolic mitral inflow velocity to mitral annular tissue velocity, *SPAP* systolic pulmonary artery pressure, *GLS* speckle tracking derived global systolic strain

## Results

Mean age of the total population was 40 ± 14 (range 10–75) years and 42.1% (70/159) of patients were male. Within the period from birth to the last follow-up visit, 38 subjects (23.9%) experienced stroke and/or TIA events (19 ischemic strokes and 35 TIAs). Stroke/TIA occurred in 19 men (27.1%, 12 ischemic strokes and 16 TIAs), and 19 women (21.3%, 7 ischemic strokes and 19 TIAs). From all individuals with cerebral events, 34 subjects had a history of stroke/TIA before baseline echocardiographic examination, 12 had recurrent stroke/TIA and 4 experienced new-onset stroke/TIA, 11 patients died (including 7 cardiac deaths) during a median of 60 (quartiles 35–90) months follow-up. All strokes were ischemic and 2 patients suffered from stroke with residual deficits.

As shown in Table 1, patients were significantly older in the events group compared to the no-events group (45 ± 13 vs. 39 ± 15 years, *P* = .020). Mean age at first onset of stroke events was 40 ± 13 (range 14–68) years. Creatinine level and the prevalence of angiokeratoma (52.6 vs. 32.2%) were significantly higher in the events group than in the no-events group (both *P* < .05). Two patients (1.7%) in the no-events group received antiplatelet medication due to coronary artery disease, and 7 patients (18.4%) in the events group received antiplatelet medication (6 with aspirin only and 1 with additional clopidogrel).

The prevalence of ST–T alterations was significantly higher in the events group than in the no-events groups (56.8 vs. 37.3%, *P* = .036). Other ECG parameters were comparable between groups. On 24-h Holter ECG monitoring, an atrial high-rate episode was documented in 2 out of 134 (1.5%) patients who did not suffer from stroke/TIA, while none was tested positive for silent AF episodes.

A total of 136 patients underwent a CMR examination (Table 1). The remaining patients (*n* = 23) did not because of the presence of contraindications to LGE imaging such as end-stage renal dysfunction, implanted cardiac pacemakers or defibrillators, or claustrophobia, etc. In 51 out of 56 (91.1%) LGE positive patients LGE positive areas were identified in the typical location at the basal inferolateral and/or inferior wall [events group *n* = 15 (100%) vs. no-events group *n* = 36 (87.8%), *P* = .309]. Right ventricular function could be measured in 133 patients by CMR with a mean RVEF of 55 ± 10%. No difference in RVEF was found comparing the no-events and events groups (54 ± 10 vs. 56 ± 11%, *P* = .312).

Fabry cardiomyopathy was identified in 80 (50.3%) patients and the proportion of cardiomyopathy was significantly higher in the events group compared to the no-events group (65.8 vs. 45.5%, *P* = .029). Definitive cardiac-related genotype mutations (N215S) were detected in 12 (7.5%) patients. The percentage of left ventricular hypertrophy (LVH) tended to be higher in these patients compared to patients without the N215S mutation [41.7% (5/12) vs. 27.2% (40/147), *P* = .322].

Echocardiography data (Table 2) showed that LV septal, LVPWd, and LV mass index were significantly higher, and GLS was remarkably lower in the events group than in the no-events group (all *P* < .05). LVEF was similar between groups (66 ± 7 vs. 65 ± 8%).

Cox regression analysis showed that history of prior stroke/TIA was the only independent predictor (adjusted HR 19.97, 95% CI 5.61–71.08, *P* < .001) while age, female sex, history of congestive heart failure, hypertension, diabetes, and vascular disease were not predictors of stroke/TIA in this Fabry cohort. Further analysis showed that elevated creatinine level (≥ 1.0 mg/dl, HR 3.74, 95% CI 1.35–10.35, *P* = .011), significant LV hypertrophy with LVPWd > 14 mm (HR 4.08, 95% CI 1.29–12.87, *P* = .017), reduced GLS (< 13.5%, HR 5.19, 95% CI 1.83–14.71, *P* = .002), and the presence of angiokeratoma (HR 4.06, 95% CI 1.41–11.69, *P* = .010) remained as independent risk predictors of stroke/TIA in FD patients (Suppl. Table 1 and Suppl. Figure 2). It is to note, although the prevalence of coronary artery disease and NYHA class were significantly higher in FD patients with stroke/TIA, Cox regression analysis showed that coronary artery disease (HR 1.063, *P* = .953) and NYHA class (HR 1.145, *P* = .858) were not independent predictors of new-onset or recurrent stroke/TIA in this patient cohort.

Based on above findings, we established a Fabry-specific score for risk stratification of stroke in FD patients (Table 3): prior stroke/TIA (2 points), angiokeratoma (1 point), creatinine ≥ 1.0 mg/dl (1 point), LVPWd > 14 mm (1 point) and GLS < 13.5% (1 point).

**Table 3:** CHA_2_DS_2_-VASc score and Fabry-specific score schemas

Risk factors	Point
CHA_2_DS_2_-VASc score	
Congestive heart failure	1
Hypertension	1
Age ≥ 75 years	2
Diabetes	1
Prior stroke/TIA	2
Vascular disease	1
Age 65–75 years	1
Female sex	1
Fabry-specific score	
Prior stroke/TIA	2
Angiokeratoma	1
Creatinine ≥ 1.0 mg/dl	1
LVPWd > 14 mm	1
GLS < 13.5%	1

*TIA* transient ischemic attack, *LVPWd* end-diastolic left ventricular posterior wall thickness, *GLS* global longitudinal strain

Supplementary Table 2 and Supplementary Fig. 3 show the incidence rates of new-onset or recurrent stroke/TIA, all-cause death, and combined events during follow-up assessed by the CHA_2_DS_2_-VASc score and Fabry-specific score in FD patients with individual risk score value. The risk of developing new-onset stroke or recurrent, all-cause mortality and combined events increased in proportion to increasing scores.

**Fig. 1:**
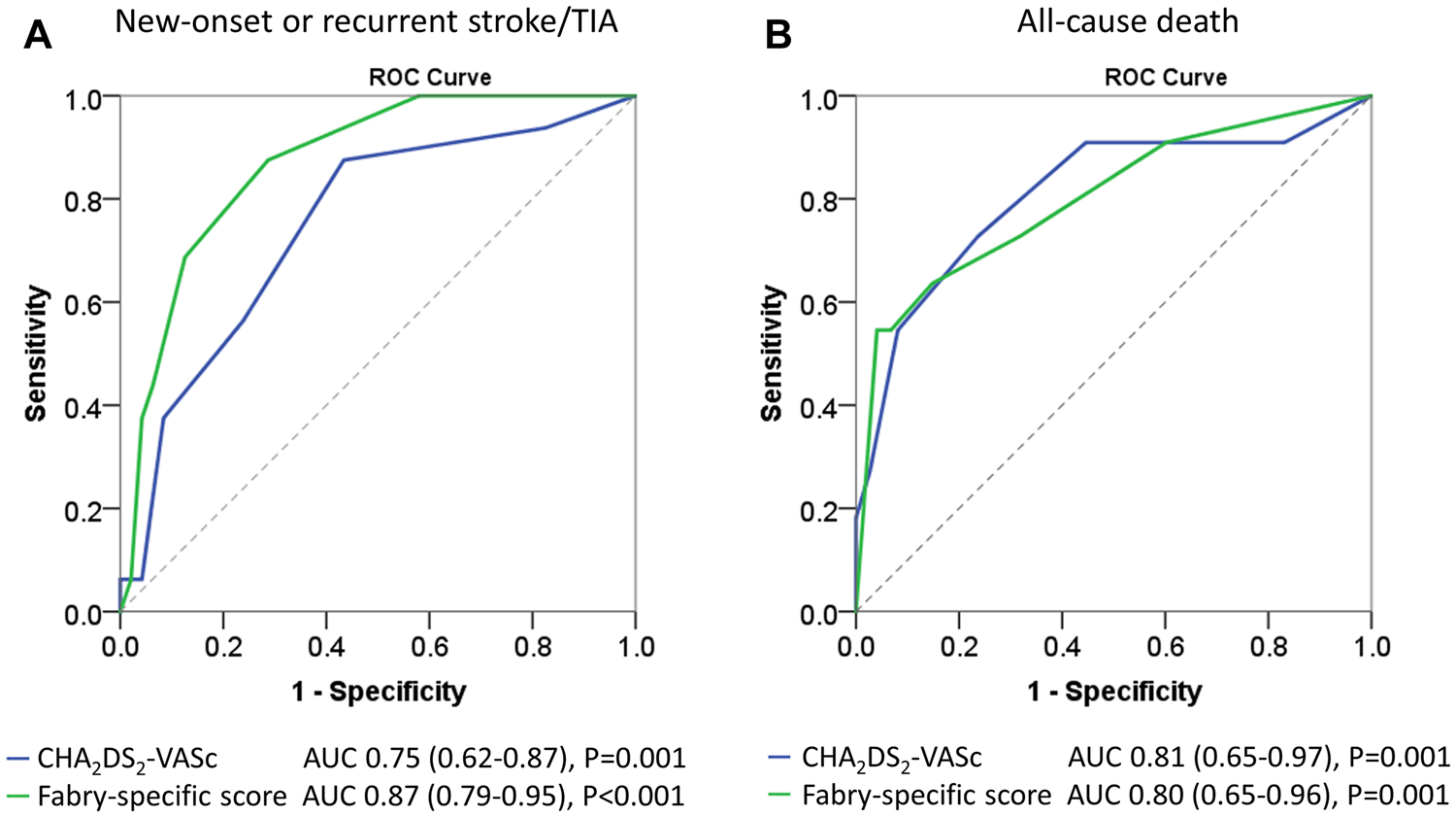
Diagnostic performance of the CHA_2_DS_2_-VASc score and the Fabry-specific score for predicting new-onset or recurrent stroke/TIA (**a**) and all-cause mortality (**b**) in Fabry patients without atrial fibrillation

There was an obvious trend that the predictive power for new-onset or recurrent stroke/TIA of the Fabry-specific score is superior to the CHA_2_DS_2_-VASc score, although this trend did not reach statistical significance in this FD cohort (AUC 0.87, 95% CI 0.79–0.94 vs. AUC 0.75, 95% CI 0.62–0.87, *P* = .199; Fig. 1a). The predicting efficacy for the all-cause mortality was comparable by both score methods (Fig. 1b).

**Fig. 2:**
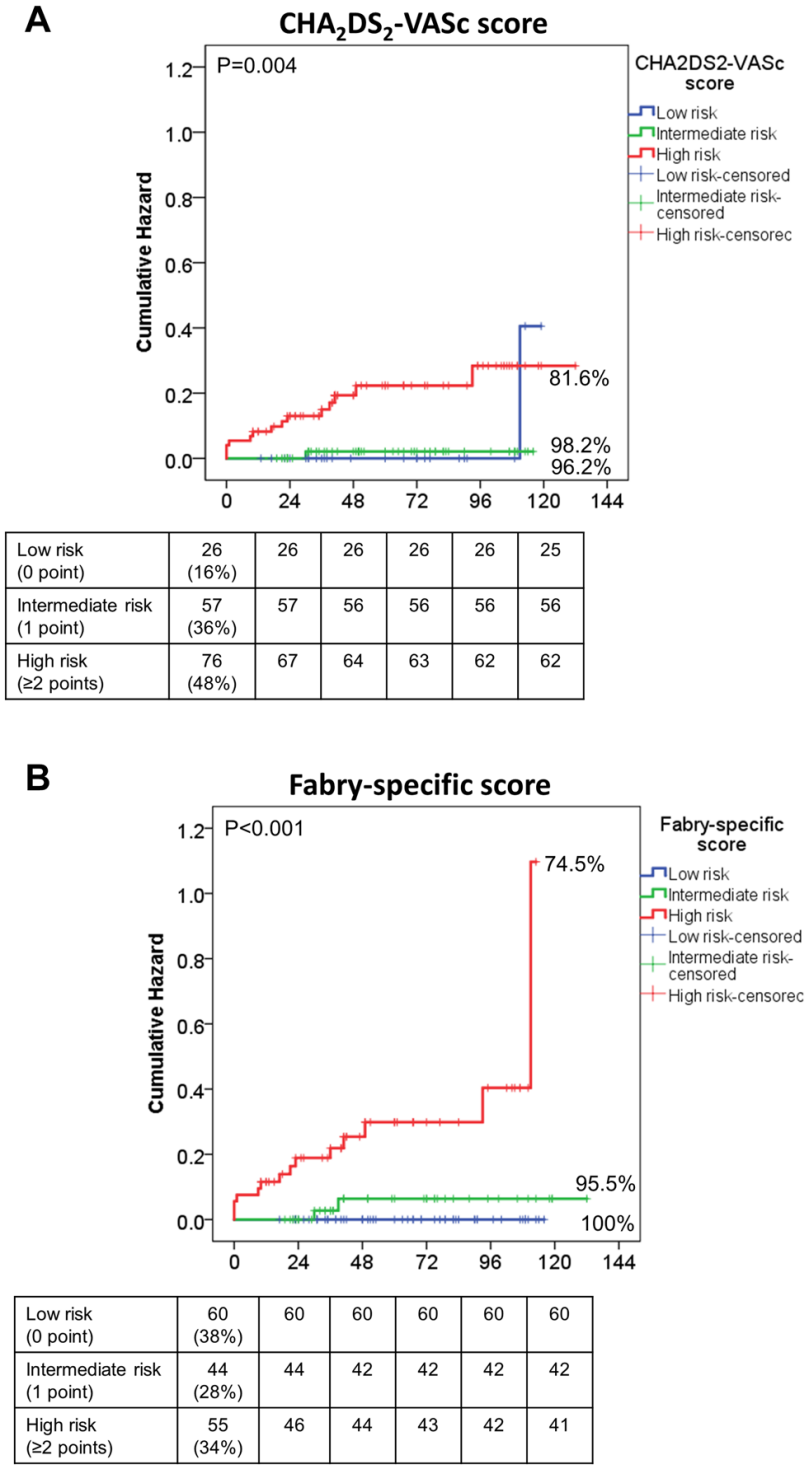
Cumulative hazard of new-onset or recurrent stroke/TIA in FD patients without atrial fibrillation stratified by low, intermediate, and high risk identified using the CHA_2_DS_2_-VASc score (**a**) and the Fabry-specific score (**b**)

As shown in Fig. 2, patients with high stroke/TIA risk (≥ 2 points) defined by both CHA_2_DS_2_-VASc score and Fabry-specific score, were associated with significantly increased incidence rate of new or recurrent stroke/TIA as compared to patients with low or intermediate risk. The annualized event rate of stroke/TIA was 0.78, 0.35 and 4.13 per 100 patient-years as assessed by the CHA_2_DS_2_-VASc score scheme for patients with low, intermediate and high risk, and was 0, 0.90 and 6.41 per 100 patient-years by Fabry-specific score scheme for patients with low, intermediate, and high risk (Suppl. Table 3).

## Discussion

The main findings of this study are, (i) the CHA_2_DS_2_-VASc score is feasible for predicting new-onset or recurrent stroke/TIA in FD patients without AF; (ii) prior stroke/TIA, angiokeratoma, creatinine ≥ 1.0 mg/dl, LVPWd > 14 mm, and GLS < 13.5% are independent risk factors for new-onset or recurrent stroke/TIA in FD patients without AF; (iii) the predicting efficacy of Fabry-specific score based on the defined risk factors in these FD patients is superior to classic CHA_2_DS_2_-VASc score on risk stratification of new-onset or recurrent stroke/TIA in FD patients without AF.

### Cerebrovascular events in FD

Cerebrovascular complication is the major cause of early morbidity and mortality in patients with FD. Ischemic stroke and TIA are the most prevalent cerebrovascular events in FD patients. Previous studies reported a wide range (from 7 to 48%) of the incidence of stroke in FD patients [18]. A large cohort study with 2446 patients in the Fabry Registry US demonstrated that the stroke incidence in FD was 6.9% in men and 4.3% in women, and increased with age [4], which was markedly higher than that in the general US population across all age categories and ∼20% of FD patients experience a first stroke below the age of 30 years [4]. The prevalence of stroke/TIA was 23.9% in our cohort and 23.7% patients experienced the first stroke with the age of < 30 years. All cerebrovascular events in our cohort were of ischemic origin, which is basically in line with previous findings in that 87% of the first stroke event was of ischemic origin [4]. The underlying pathogenic mechanisms of stroke in Fabry disease remain elusive now, possibly linked with an interaction between the progressive neuronal accumulation of glycosphingolipids and specific cellular proteins, leading to cellular and organ dysfunction [19]. Lysosomal accumulations of GL-3 could induce oxidative stress and promote the formation of reactive oxygen species, thus causing sustained dilation of the cerebral vessels, which might result in cerebral vasculature endothelial dysfunction and increase the vulnerability of cerebral vasculature [19, 20]. Cerebral blood vessel abnormalities in FD are characterized by cerebral glucose metabolism impairment due to endothelial proliferation or vessel stenosis. Increased platelet reactivity and altered cerebrovascular reactivity because of autonomic dysfunction might also attribute to vasculopathy of stroke in FD [21].

### Risk assessment of stroke/TIA in FD patients using the CHA_2_DS_2_-VASc risk score

The CHA_2_DS_2_-VASc score is feasible to predict stroke risk in heart failure patients without AF. Our study results demonstrated for the first time that this score is also useful for predicting new-onset stroke risk in FD patients without AF (AUC 0.749, 95% CI 0.624–0.874, *P* = .001). The most powerful contributing element among all co-risk factors, however, was the presence of prior stroke history, while additional risk factors including age and gender only played a minor role in this FD entity.

### Risk assessment of stroke/TIA in FD patients by the Fabry-specific score

Previous stroke/TIA was included in this Fabry-specific score, since this is a major contributing factor for risk assessment of new-onset or recurrent stroke.

Another major player in the Fabry-specific score is the presence of angiokeratoma of FD patients. Angiokeratoma is a common and early dermatological manifestation in FD. In the early years, term of “Angiokeratoma corporis diffusum” was used to define Fabry disease [22, 23]. It may be the earliest physical sign of FD, and often occurs in children between the ages of 5 and 15 years of Fabry patients [24]. Angiokeratoma is a kind of benign skin lesion characterized by proliferation of dilated blood vessels in the upper dermis, caused by accumulation of Gb3 in dermal endothelial cells, followed by vascular ectasia [25, 26]. Information regarding the prevalence of angiokeratoma in FD remains largely limited. Larralde et al. reported a quite high angiokeratoma incidence of ∼80% in FD [24]. A retrospective clinical and CMR study with a small cohort (*n* = 43) also revealed a high incidence of angiokeratoma (88%) in male FD patients [27]. In contrast, angiokeratoma was detected in only one (1%) adult female FD patient (< 40 years) in a recent clinical study with a cohort of 191 adult and pediatric Fabry patients carrying the IVS4 + 919G > A mutation [28]. Data from 261 adult female FD patients from 6 German Fabry centers showed that 39% of female FD patients had angiokeratoma. In female FD patients, angiokeratoma is more frequently documented in patients with nonsense mutations as compared to patients with missense mutations (61 vs. 40%, *P* < .05) [29]. In our cohort, angiokeratoma is documented in one-third of FD patients (total 37%, 19% in female vs. 60% in male, *P* < .001), and the prevalence of angiokeratoma is significantly higher in patients with stroke/TIA than in those without stroke/TIA (52.6 vs. 32.2%, *P* = .023), especially in male patients with stroke/TIA than in those without stroke/TIA (90 vs. 49%, *P* = .002). Results from our group and others thus indicate that prevalence of angiokeratoma in FD patients varies significantly depending on mutations in the GLA gene and gender and this skin lesion occurs more commonly in men with FD than in women with FD. A previous study from our group found that this gender difference in prevalence of angiokeratoma may be related to the higher distal skin Gb3 load in male FD patients as compared to female FD patients and to health controls based on skin biopsy results [30]. Due to the close association between the presence of angiokeratoma and stroke/TIA events in our FD cohort, and the finding that angiokeratoma served as an independent factor of new-onset or recurrent stroke/TIA based on the results of the multivariable Cox regression models, angiokeratoma is included in the Fabry-specific score. To our knowledge, this is the first report on the association between angiokeratoma and stroke risk in Fabry disease. Although this skin lesion is not non-specific for Fabry disease, pathologically, the underlying mechanisms are both related to accumulation of Gb3 in dermal vasculature or cerebral vasculature, thus, it is tempting to speculate that visible skin angiokeratoma might be linked with stroke/TIA due to the common pathological changes of the dermal and cerebral vasculature.

STI derived global systolic strain (GLS) has been extensively applied to assess and monitor LV systolic global function as well as to predict outcome in various cardiovascular disorders in recent years [31–33]. Previous studies have demonstrated that GLS could provide additional information on cerebrovascular risk burden [34, 35]. In line with above finding, results from the multivariable Cox regression models in our FD cohort also indicated that reduced (< 13.5%) GLS value is capable of predicting the risk of new-onset or recurrent stroke/TIA and is associated with about fivefold increased stroke/TIA risk than GLS ≥ 13.5% in our FD cohort. It is thus reasonable to include this fundamental parameter reflecting LV systolic function in our Fabry-specific score.

LVH has been recognized as a risk factor for stroke [36–39]. Likewise, a CMR study revealed that the LV size was related to stroke in a multiethnic cohort, suggesting that concentric ventricular remodeling predicted incident stroke [40]. Left ventricular concentric hypertrophy is a common imaging finding in Fabry disease and is reported in up to 50% of males and one-third of females. In our cohort, significant LVH (LVPWd > 14 mm) is detected in 26% of patients with stroke/TIA (37% of males vs. 16% of females, *P* = .269), whereas detected in about 4% of patients without stroke/TIA (7% of males vs. 4% of females, *P* = .459), suggesting that significant LVH serves as a potential risk factor for new-onset or recurrent stroke/TIA in patients with FD.

Renal involvement is a typical disease characteristic of FD patients. The link between chronic kidney disease and cerebrovascular disease has become more apparent [41]. Our results also showed that the creatinine level was significantly higher in the events group than in the no-events group and creatinine ≥ 1.0 mg/dl serves as an independent predictor of new-onset or recurrent stroke/TIA in FD patients without AF.

### Predicting efficacy of the risk of new-onset or recurrent stroke/TIA by the Fabry-specific score

In the present study, we explored the potential role of easy-to-obtain clinical [Fabry associated skin markers (angiokeratoma), renal dysfunction (elevated creatinine), and prior stroke/TIA] and echocardiographic parameters (LVPWd and GLS) in new-onset or recurrent stroke/TIA risk assessment of FD patients. The new score presents more precise predictive performance for new-onset or recurrent stroke/TIA risk assessment in FD. Diagnostic power of high risk ≥ 2 points according to the Fabry-specific score for predicting new-onset or recurrent stroke/TIA was: specificity 71%, sensitivity 88%; while the specificity was 57%, sensitivity was 88% according to the CHA_2_DS_2_-VASc risk score. In addition, 38% of Fabry patients were identified as low stroke risk, 28% as intermediate risk, and 34% as high risk (≥ 2 points) according to the Fabry-specific score. In contrast, these values assessed by CHA_2_DS_2_-VASc score were 16, 36 and 48%, respectively. Thus, the Fabry-specific score fairly well represents the real-world risk scenario in this FD cohort for new-onset or recurrent stroke/TIA, while the CHA_2_DS_2_-VASc score underestimated the FD patients with low stroke risk and overestimated the FD patients with high stroke risk. AUC values of Fabry-specific score (AUC 0.87) is also superior to the classic CHA_2_DS_2_-VASc score (AUC 0.75).

In the present study, prior events of TIA were included in the Fabry-specific score as a criterion with two points and compared with the CHA_2_DS_2_-VASc score for the predictive efficacy on risk of stroke/TIA in this patient cohort. The predicting efficacy was better using the Fabry-specific score than the CHA_2_DS_2_-VASc score in this study setting; however, the CHA_2_DS_2_-VASc score was originally developed to predict stroke events and not TIA. This difference in the two scores needs to be kept in mind when interpreting the current data analysis.

### Clinical implication

Despite manifold reasons for cerebro- or cardiovascular events have been revealed until today, predictability and optimum treatment remain controversial [42–46], stressing the need for further research in this regard. Our results demonstrate that the Fabry-specific score is useful for the risk assessment of new-onset or recurrent stroke/TIA in FD patients without AF. Since all new-onset or recurrent stroke/TIA events in this cohort were of ischemic origin, and the specificity and sensitivity for predicting new-onset or recurrent stroke/TIA by the high risk (Fabry score ≥ 2 points) were around 70–80%. It is therefore reasonable to monitor FD patients based on this score, since those with high calculated risk might benefit from additional therapy such as antithrombotic medication in an effort to reduce future events of new-onset or recurrent stroke/TIA in FD patients.

### Study limitations

The Fabry-specific stroke risk assessment is established on the risk factors defined in a single-center cohort with a relatively small sample size. The predicting efficacy of the new Fabry-specific score should be tested and validated in other FD cohort. Furthermore, future multicenter registry studies are needed to define the efficacy and safety of antiplatelet or anticoagulant regimens in FD patients with and without AF.

## Conclusion

Prior stroke/TIA, angiokeratoma, LVPWd > 14 mm, creatinine ≥ 1.0 mg/dl and GLS < 13.5% are independent risk factors for new-onset or recurrent stroke/TIA in FD patients without AF. The new predicting score based on the above defined risk factors in this FD patient cohort is superior to classic CHA_2_DS_2_-VASc score on predicting new-onset or recurrent stroke/TIA in FD patients without AF. FD patients with high risk for new-onset or recurrent ischemic stroke/TIA might therefore benefit from the regular and intensive antithrombotic therapy.

## Electronic supplementary material

Below is the link to the electronic supplementary material.

Supplementary material 1 (DOCX 12 KB)

Supplementary material 2 (TIF 215 KB)

Supplementary material 3 (TIF 1206 KB)

Supplementary material 4 (TIF 694 KB)

Supplementary material 5 (PDF 92 KB)

Supplementary material 6 (PDF 92 KB)
