# Supplementary material for: Value of the CHA2DS2-VASc score and Fabry-specific score for predicting new-onset or recurrent stroke/TIA in Fabry disease patients without atrial fibrillation
Source: Clin Res Cardiol. 2018 May 24;107(12):1111–21. doi: 10.1007/s00392-018-1285-4 (PMC6244978; doi:10.1007/s00392-018-1285-4)
Supplement: Supplementary file 1 — Supplementary material 1 (DOCX 12 KB) [file 392_2018_1285_MOESM1_ESM.docx]

**Supplementary figures legends**

**Suppl. Figure 1. Flow-chart of the study protocol.** FAZIT: the Fabry Center for Interdisciplinary Treatment; FD: Fabry disease; STI: speckle tracking imaging; AF: atrial fibrillation; CI: confidence interval.

**Suppl. Figure 2. Cumulative hazard of new-onset or recurrent stroke/TIA in Fabry patients without atrial fibrillation stratified by related risk factors including prior stroke/TIA history (A), angiokeratoma (B), creatinine (C), LVPWd (D), or GLS (E).** LVPWd: the wall thickness of left ventricular posterior wall; GLS: global longitudinal strain. HR: hazard ratio; CI: confidence interval.

**Suppl. Figure 3. Incidence rates of new-onset or recurrent stroke/TIA, all-cause death, and combined events during follow-up assessed by the CHA_2_DS_2_-VASc score (A) and the Fabry-specific score (B) in Fabry patients with respective risk score.**
