# Supplementary figures and images for: Value of the CHA2DS2-VASc score and Fabry-specific score for predicting new-onset or recurrent stroke/TIA in Fabry disease patients without atrial fibrillation
Source: Clin Res Cardiol. 2018 May 24;107(12):1111–21. doi: 10.1007/s00392-018-1285-4 (PMC6244978; doi:10.1007/s00392-018-1285-4)

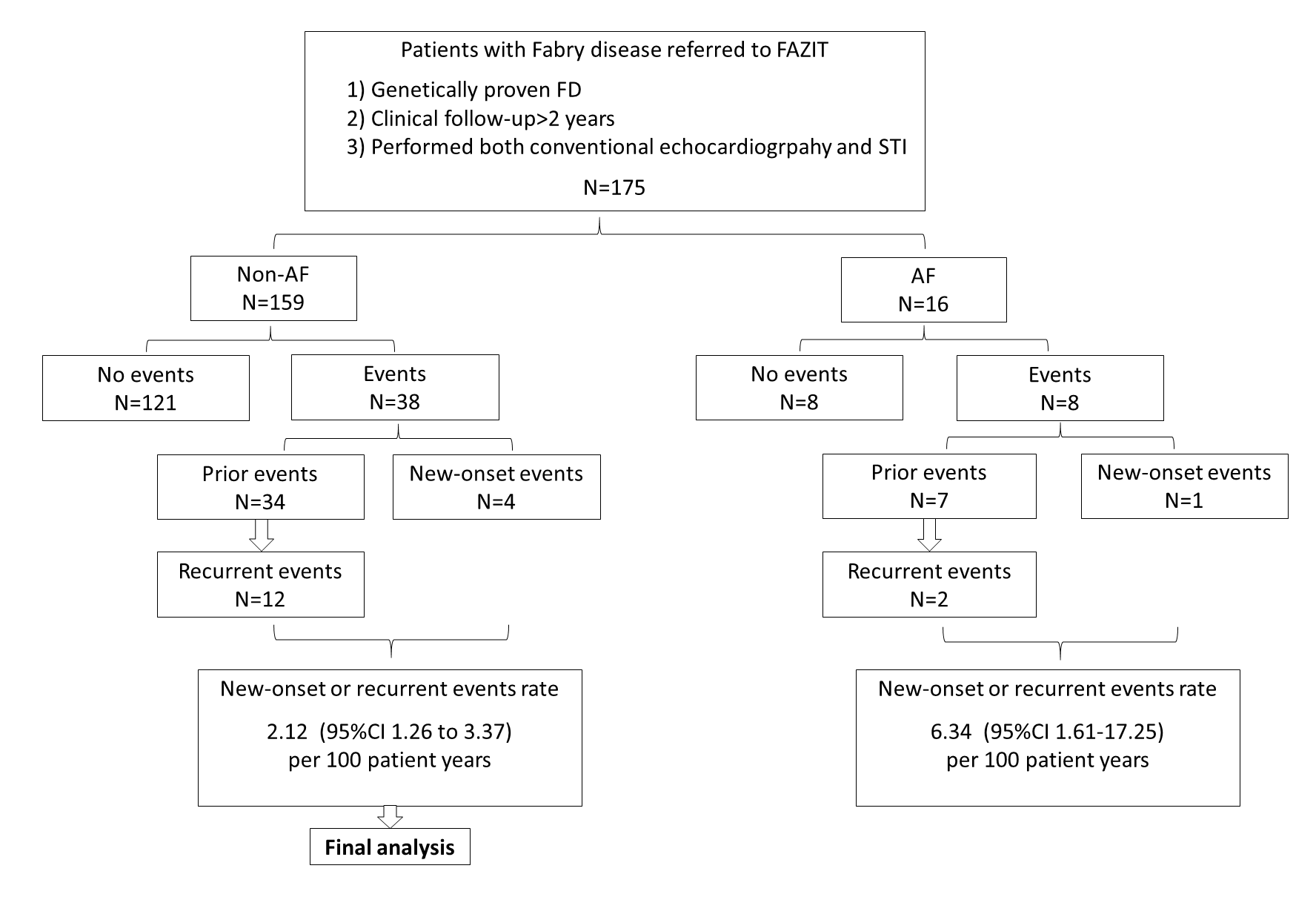

Supplement: Supplementary file 2 — Supplementary material 2 (TIF 215 KB) [file 392_2018_1285_MOESM2_ESM.tif]

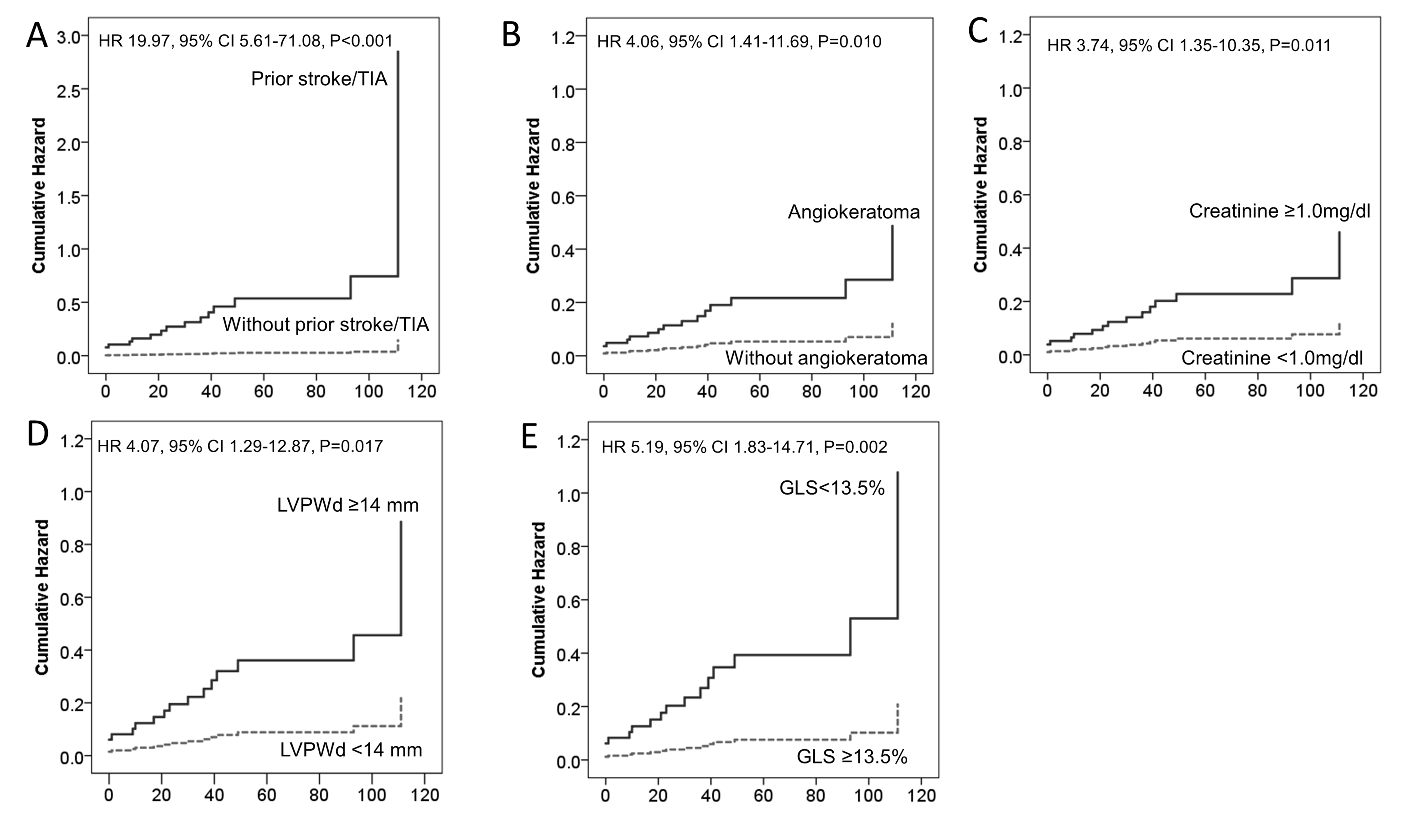

Supplement: Supplementary file 3 — Supplementary material 3 (TIF 1206 KB) [file 392_2018_1285_MOESM3_ESM.tif]

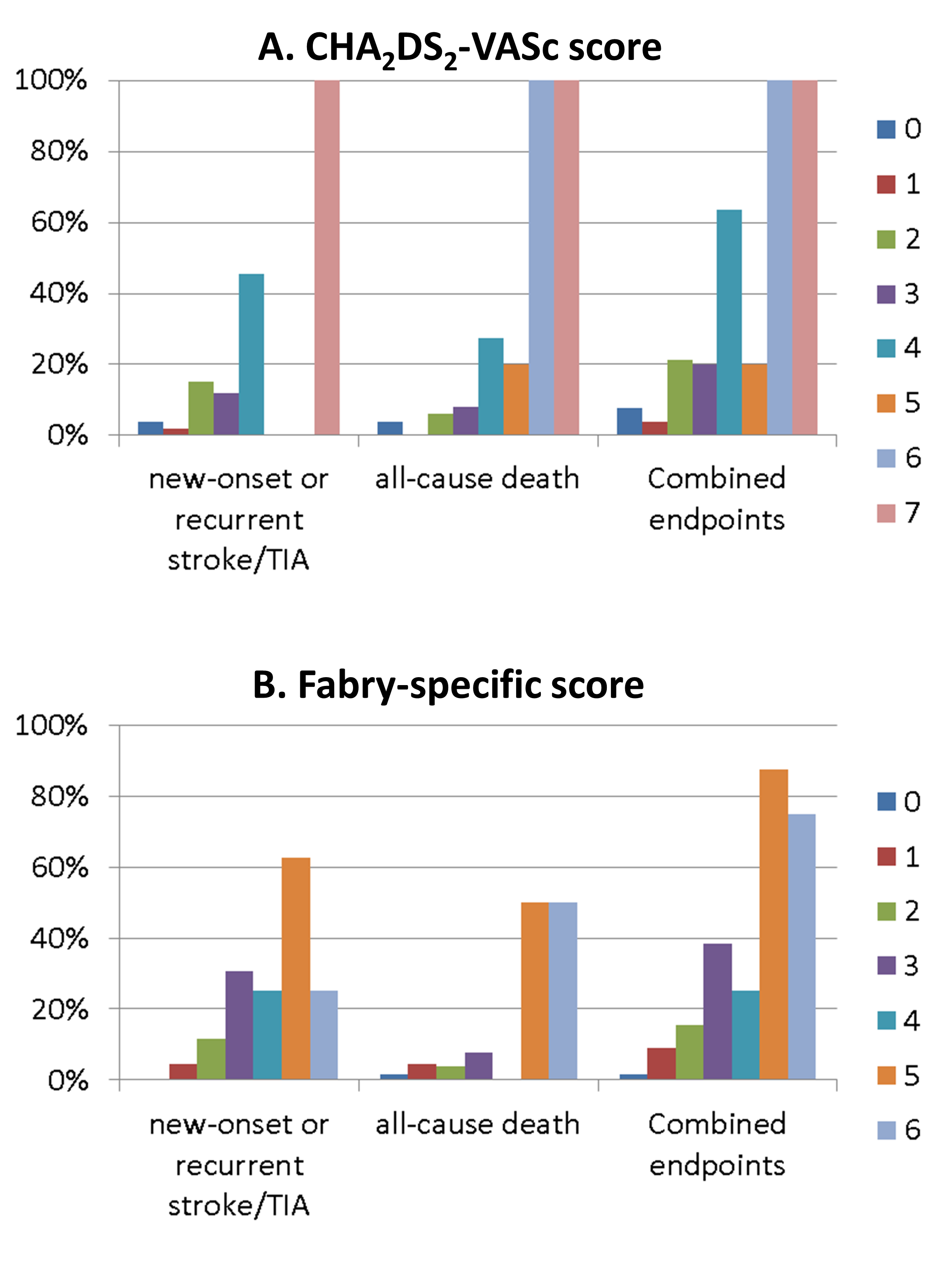

Supplement: Supplementary file 4 — Supplementary material 4 (TIF 694 KB) [file 392_2018_1285_MOESM4_ESM.tif]
