# Supplementary material for: Value of the CHA2DS2-VASc score and Fabry-specific score for predicting new-onset or recurrent stroke/TIA in Fabry disease patients without atrial fibrillation
Source: Clin Res Cardiol. 2018 May 24;107(12):1111–21. doi: 10.1007/s00392-018-1285-4 (PMC6244978; doi:10.1007/s00392-018-1285-4)
Supplement: Supplementary file 5 — Supplementary material 5 (PDF 92 KB) [file 392_2018_1285_MOESM5_ESM.pdf]

Supplemental Table 1 Hazard ratio of risk factors for predicting new-onset stroke/TIA in FD patients without AF

	New-onset or recurrent stroke/TIA		
	**Hazard ratio**	**95% CI**	**P value**
**Univariable**			
**CHA_2DS_2-VASc Score**			
**Age at baseline**	1.020	0.984-1.056	0.276
**Female sex**	0.613	0.227-1.651	0.333
**NYHA class II-IV**	1.145	0.259-5.062	0.858
**Hypertension**	2.137	0.800-5.711	0.130
**Prior stroke/TIA**	19.967	5.609-71.082	<0.001
**Vascular disease**	1.063	0.140-8.093	0.953
**Diabetes**	7.606	0.991-58.390	0.051
**Fabry associated risk factors**			
**Angiokeratoma**	4.057	1.407-11.694	0.010
**Cornea verticillata**	1.891	0.465-7.690	0.373
**Creatinine ≥1.0mg/dl vs. <1.0mg/dl**	3.741	1.352-10.349	0.011
**LVPWd (mm)**	1.272	1.085-1.491	0.003
**LVPWd >14mm vs. ≤14mm**	4.075	1.291-12.867	0.017
**LAVi (ml)**	1.009	0.957-1.063	0.748
**E/e´**	1.029	0.966-1.096	0.371
**LVEF (%)**	1.019	0.948-1.095	0.606
**GLS (%)**	1.124	1.023-1.236	0.015
**GLS <13.5% vs. ≥13.5%**	6.019	2.295-15.787	<0.001
**Multivariable adjusted for age, sex, and coronary artery disease**			
**Prior stroke/TIA**	19.967	5.609-71.082	<0.001
**Angiokeratoma**	4.057	1.407-11.694	0.010
**Creatinine ≥1.0mg/dl vs. <1.0mg/dl**	3.741	1.352-10.349	0.011
**LVPWd >14mm vs. ≤14mm**	4.075	1.291-12.867	0.017
**GLS <13.5% vs. ≥13.5%**	5.188	1.830-14.710	0.002

TIA: transient ischemic attack; AF: atrial fibrillation; NYHA: New York Heart Association; LVPWd: end-diastolic left ventricular posterior wall thickness**;** LAVi: left atrial volume indexed to body surface area; E/e´: the ratio of early diastolic mitral inflow velocity to mitral annular tissue velocity; LVEF: left ventricular ejection fraction; GLS: speckle tracking derived global systolic strain.

Supplemental Table 2 Incidence rates of new-onset cerebrovascular events and all-cause death in patients with Fabry disease during follow-up

Endpoints		CHA_2DS_2-VASc score							
	Events/ Total No.	0	1	2	3	4	5	6	7
New-onset or recurrent stroke/TIA	16/159	1/26	1/57	5/33	3/25	5/11	0/5	0/1	1/1
	10.1%	3.8%	1.8%	15.2%	12.0%	45.5%	0.0%	0.0%	100%
All-cause death	11/159	1/26	0/57	2/33	2/25	3/11	1/5	1/1	1/1
	10.3%	3.8%	0.0%	6.1%	8.0%	27.3%	20.0%	100%	100%
Combined endpoints	25/159	2/26	1/57	7/33	5/25	7/11	1/5	1/1	1/1
	15.7%	7.7%	4.0%	21.2%	20.0%	63.6%	20.0%	100%	100%

		Fabry-specific score						
	Events/ Total No.	0	1	2	3	4	5	6
New-onset or recurrent stroke/TIA	16/159	0/60	2/44	3/26	4/13	1/4	5/8	1/4
	10.1%	0.0%	4.5%	11.5%	30.8%	25.0%	62.5%	25.0%
All-cause death	11/159	1/60	2/44	1/26	1/13	0/4	4/8	2/4
	6.9%	1.7%	4.5%	3.8%	7.7%	0%	50.0%	50.0%
Combined endpoints	25/159	1/60	4/44	4/26	5/13	1/4	7/8	3/4
	15.7%	1.7%	9.1%	15.4%	38.5%	25.0%	87.5%	75.0%
